# Assessment of four organophosphorus pesticides as inhibitors of human acetylcholinesterase and butyrylcholinesterase

**DOI:** 10.1038/s41598-021-00953-9

**Published:** 2021-11-02

**Authors:** Tena Čadež, Dora Kolić, Goran Šinko, Zrinka Kovarik

**Affiliations:** Institute for Medical Research and Occupational Health, Ksaverska cesta 2, 10 000 Zagreb, Croatia

**Keywords:** Enzymes, Biochemistry, Enzyme mechanisms, Computational neuroscience, Regeneration and repair in the nervous system, Toxicology

## Abstract

Toxicity of organophosphorus compounds (OPs) remains a major public health concern due to their widespread use as pesticides and the existence of nerve agents. Their common mechanism of action involves inhibition of enzymes acetylcholinesterase (AChE) and butyrylcholinesterase (BChE) which are crucial for neurotransmission. Both chronic and acute poisoning by OPs can leave long-lasting health effects even when the patients are treated with standard medical therapy. Therefore, an increasing urgency exists to find more effective oxime reactivators for compounds which are resistant to reactivation, especially phosphoramidates. Here, we investigated in silico and in vitro interactions and kinetics of inhibition for human cholinesterases with four organophosphate pesticides—ethoprophos, fenamiphos, methamidophos and phosalone. Overall, ethoprophos and fenamiphos displayed higher potency as inhibitors for tested cholinesterases. Our results show that methamidophos-inhibited hAChE was more susceptible to reactivation than hAChE inhibited by fenamiphos by selected oximes. Molecular modelling enabled an evaluation of interactions important for specificity and selectivity of both inhibition and reactivation of cholinesterases. Two newly developed reactivators—bispyridinium triazole oxime 14A and zwitterionic oxime RS194B possess remarkable potential for further development of antidotes directed against pesticides and related phosphoramidate exposures, such as nerve agents tabun or Novichoks.

## Introduction

Organophosphorus (OP) compounds are widely used in today’s world and can be a cause of great risk for people. Chemical weapons, known as nerve agents, mostly take the main role in discussions of organophosphorus compounds while organophosphorus pesticides are often easily overlooked even though they cause more than 3 million poisonings cases a year^1^. OPs are among the most common synthetic chemicals detected in rivers, groundwater, soil, air and plants, upholding them as an increasing environmental concern. Residual presence of pesticides in a variety of fruits, vegetables and agricultural crops nowadays can often exceed maximum residue limits due to their bioaccumulation and persistence in the ecosystem^2,3^. Exposure to organophosphorus pesticides through direct contact, ingestion or inhalation can have a deleterious effect on human health; additional residue in food also contributes to the health risk^4,5^. Such exposure can lead to distinct neurotoxic effects depending on dose, frequency and route of exposure^6,7^. The main action mechanism of OP compounds is inhibition of cholinergic enzymes acetylcholinesterase (AChE) and butyrylcholinesterase (BChE), causing accumulation of neurotransmitter acetylcholine in the synaptic cleft and inducing an excessive stimulation of nicotinic and muscarinic receptors in the central and peripheral nervous system, leading to a paralysis of cholinergic synaptic transmission^8,9^. Organophosphate poisoning ultimately results in cholinergic crisis and a variety of symptoms such as miosis, bronchoconstriction, bradycardia, excessive salivation, muscle fasciculations, respiratory depression, loss of consciousness and epileptic seizures^6,8–10^. Even when exposed individuals receive proper medical care, long-lasting neurological, behavioural and cognitive changes can occur due to excitotoxic damage to the central nervous system^10–12^.

**Figure 1:**
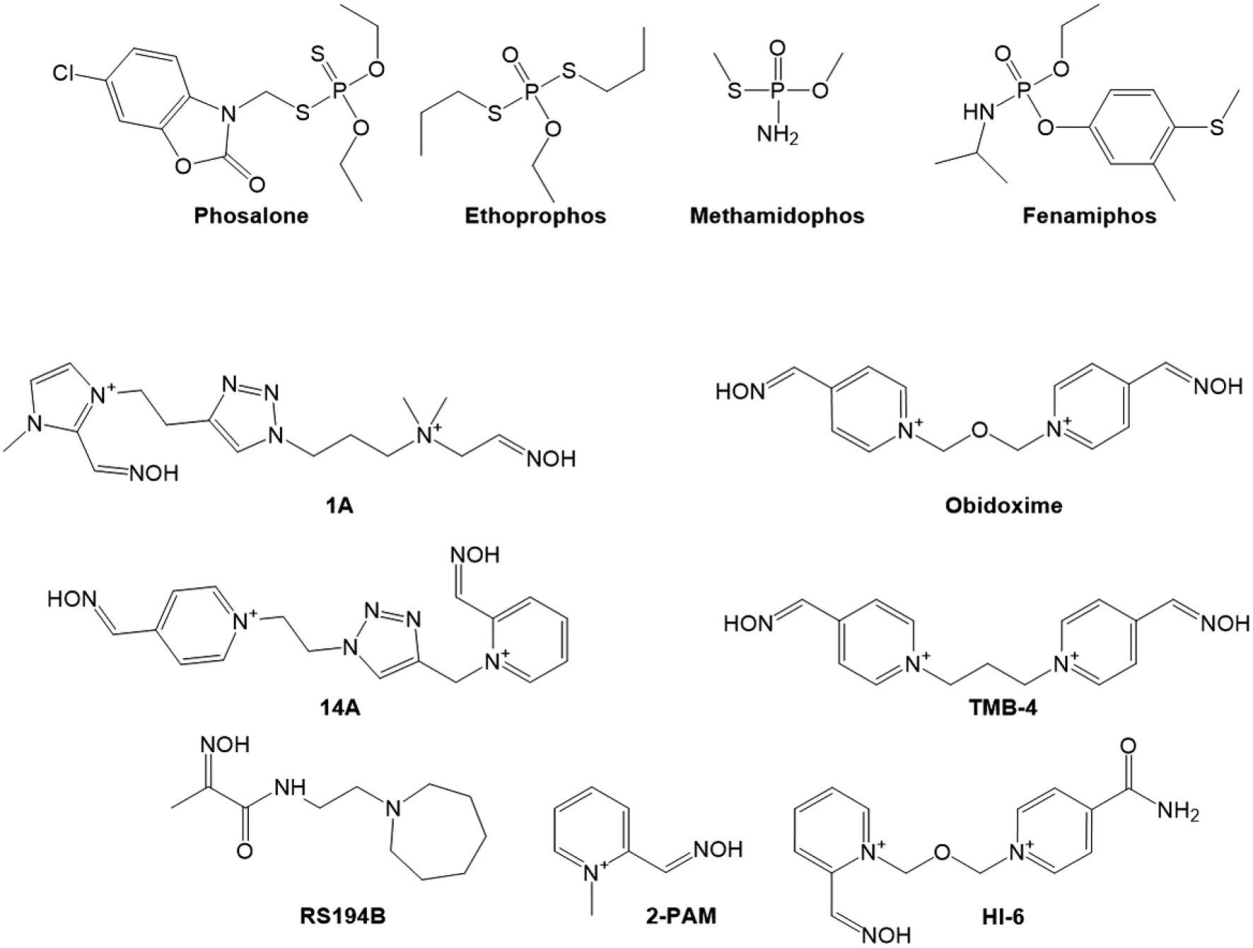
Structure of organophosphorus pesticides and selected oximes used in this study.

Organophosphorus pesticides, as methamidophos, fenamiphos, phosalone and ethoprophos (Fig. 1), are recognized as potent inhibitors of AChE that can affect the nervous system by interfering with normal nerve impulse transmission^13–16^. Majority of pesticides as phosalone and ethoprophos (Fig. 1) are thio-phosphates that undergo biotransformation via oxidation to their usually more toxic oxo-phosphate form^12,17^. Methamidophos and fenamiphos are phosphoramidates that already have oxo-phosphate form, which could make them effective cholinergic inhibitors similar to their analogue, a well-known nerve agent tabun^14^. To successfully prevent cholinergic crisis and avoid severe health effects after exposure to OPs, prompt dephosphylation of AChE active centre needs to be done by compounds with strong nucleophile such as an oxime group^18,19^. The current medically approved oxime-antidotes (Fig. 1) were shown to be particularly ineffective for reactivation of phosphoramidated AChE, and in addition, as permanently charged compounds they do not cross the blood–brain barrier (BBB) at therapeutically relevant levels^19,20^.

In this study, we evaluate inhibition of cholinesterase activity with four organophosphorus pesticides both in silico and in vitro with an aim to identify their interactions with active site residues of both cholinesterases. A focus of this paper is also reactivation of pesticide-inhibited AChE with two click chemistry-synthetized quaternary oximes 1A and 14A that were proven to be the most efficient reactivators of nerve agent tabun-inhibited AChE which also belongs to the class of phosphoroamidates, same as methamidophos and fenamiphos^21^. Along with the quaternary oximes we test a zwitterionic hydroxyimino-acetamido alkylamine oxime, RS194B, that has been shown as an effective in vitro reactivator of human AChE and antidote against insecticide paraoxon exposure (Fig. 1). Due to its ionizable structure having four species of different ionization states, the neutral species partitions into the capillary endothelial cell and crosses BBB resulting in rapid reactivation of OP-inhibited AChE in central nervous system^22–25^.

## Results

### Progressive inhibition of cholinesterases

**Figure 2:**
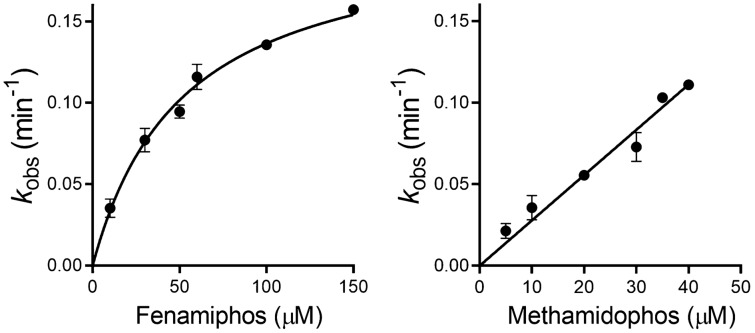
Progressive inhibition of recombinant human AChE with fenamiphos and methamidophos determined at 25 °C. Values are expressed as mean ± SEM (n = 7).

Kinetic constants of phosphorylation of AChE and BChE were determined to delineate the impact of OP pesticides on the cholinergic enzymes. Enzyme activity was measured at a range of inhibitor concentrations to determine the overall second-order rate constant of inhibition (*k*_i_), while in case of the non-linear relationship we determined intrinsic inhibition constants: the first-order inhibition constant (*k*_max_) and enzyme-inhibitor equilibrium dissociation constant (*K*_i_). The kinetic constants for the progressive inhibition of human hAChE and hBChE with selected pesticides are summarized in Table 1. Overall, ethoprophos and fenamiphos displayed the highest inhibition for both cholinesterases. A saturation curve was observed only for with fenamiphos-inhibited hAChE (Fig. 2) that enabled evaluation of *k*_max_ and *K*_i_. However, the most potent hAChE inhibitor was ethoprophos, whose bimolecular rate constant of inhibition, *k*_i_, was approximately 30-fold higher than that of phosalone and methamidophos, and 16-fold above the rate for fenamiphos (Table 1).

**Table 1:** Constants (± SEM) for inhibition of human acetylcholinesterase (hAChE) and human butyrylcholinesterase (hBChE) by selected pesticides.

Pesticide	*k*_max_ (min^-1^)	*K*_i_ (µM)	*k*_i_ (min^-1^ M^-1^)
**hAChE**			
Phosalone	/	/	2,133 ± 88
Ethoprophos	/	/	64,940 ± 2,114
Methamidophos	/	/	2,760 ± 119
Fenamiphos	0.21 ± 0.01	52.12 ± 6.19	3,982 ± 264
**hBChE**			
Phosalone	0.23 ± 0.02	97.90 ± 15.34	2,332 ± 166
Ethoprophos	0.58 ± 0.05	36.42 ± 6.68	15,840 ± 1,658
Methamidophos	/	/	386 ± 16
Fenamiphos	0.16 ± 0.01	4.38 ± 1.21	35,890 ± 8,484

The first-order inhibition constant (*k*_max_), enzyme-inhibitor equilibrium dissociation constant (*K*_i_) and the overall second-order rate constant of inhibition (*k*_i_) were determined from *k*_obs_ constants obtained from at least three experiments at 25 °C.

In case of hBChE, although all selected OP pesticides exhibited potency to progressively inhibit enzyme activity, methamidophos had the lowest potency with *k*_i_ 90-fold lower than that of fenamiphos (Table 1). Based on the intrinsic inhibition constants, it seemed that binding affinity, reflected in *K*_i_, governs the specificity of BChE inhibition. In other words, fenamiphos with the highest binding affinity was the most potent inhibitor of hBChE, while ethoprophos followed as the second strongest inhibitor even with the highest *k*_max_.

### Molecular modelling of pesticide − ChE complex

Molecular modelling of Michaelis–Menten type complex of the OP pesticides within the active site of human ChEs enabled us to visualise interactions between pesticide molecule and amino acids lining the active site gorge. The main criteria for selection of docking position for both AChE or BChE was orientation of pesticide molecule in which the leaving group of pesticide is oriented opposite from oxygen (Oγ) of ChE catalytic serine. This orientation is in accordance with a nucleophilic attack by the catalytic serine during enzyme progressive inhibition.

**Figure 3:**
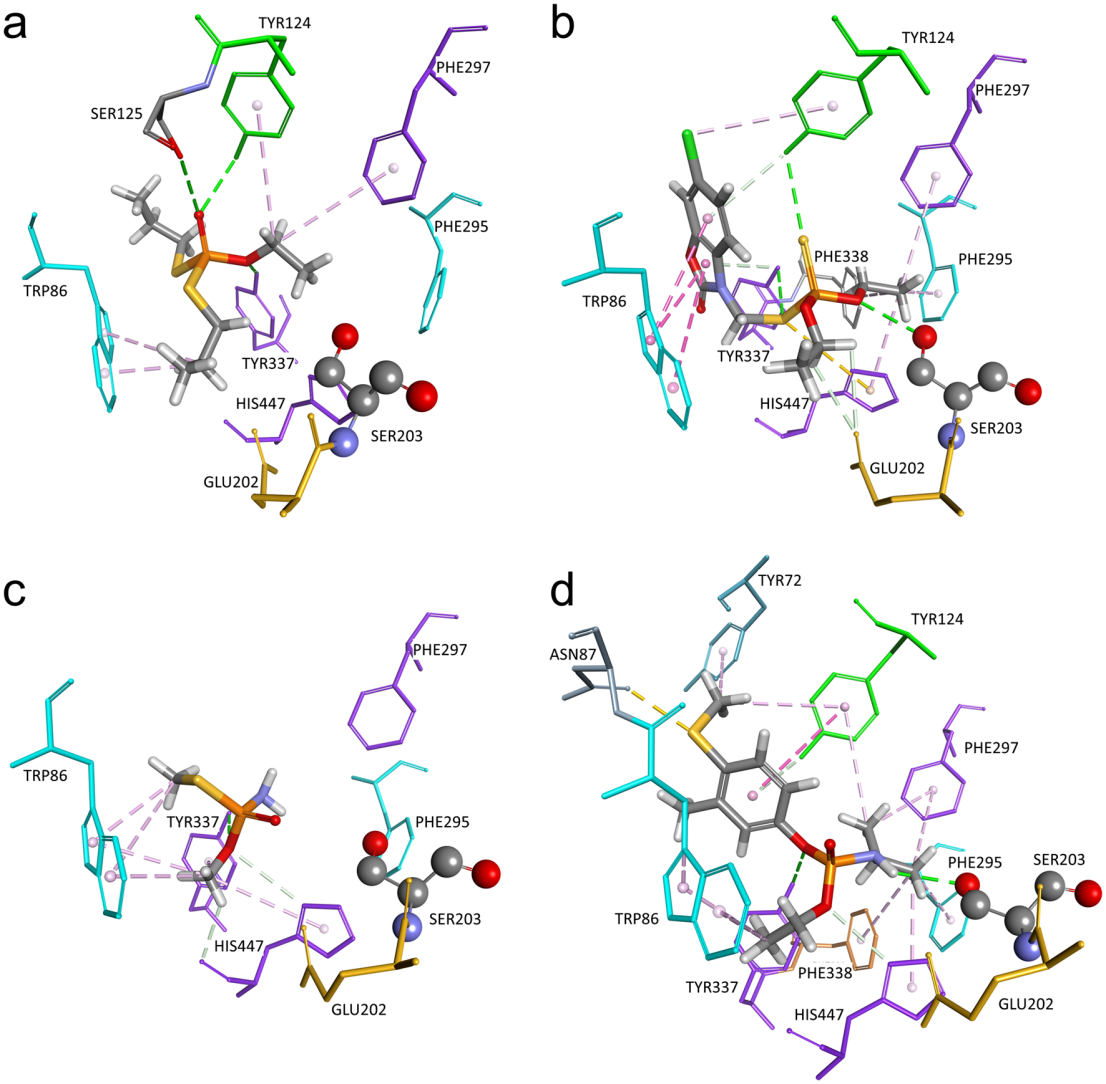
Model of Michaelis–Menten type complex of organophosphorus pesticides: ethoprophos (**a**), phosalone (**b**), methamidophos (**c**), and fenamiphos (**d**) within the active site of hAChE (PDB code: 4PQE^52^). Poses were selected based on criteria that leaving group of pesticide is oriented opposite from the oxygen (Oγ) of catalytic serine (Ser203). Pesticide interactions are represented as dashed lines: hydrophobic (purple), hydrogen bonds (green) and electrostatic (orange).

Since pesticides vary in molecular structure, one can see difference in hAChE active site residues interactions with selected pesticides (Fig. 3). Model of pesticide-AChE complex revealed formation of hydrophobic interactions and hydrogen bonds. Trp86 located at the choline binding site that stabilises substrate molecule during catalytic turnover also creates interactions with pesticides during progressive inhibition. Pesticides phosalone and fenamiphos are positioned near catalytic Ser203 and therefore create the hydrogen bond with Ser203.

**Figure 4:**
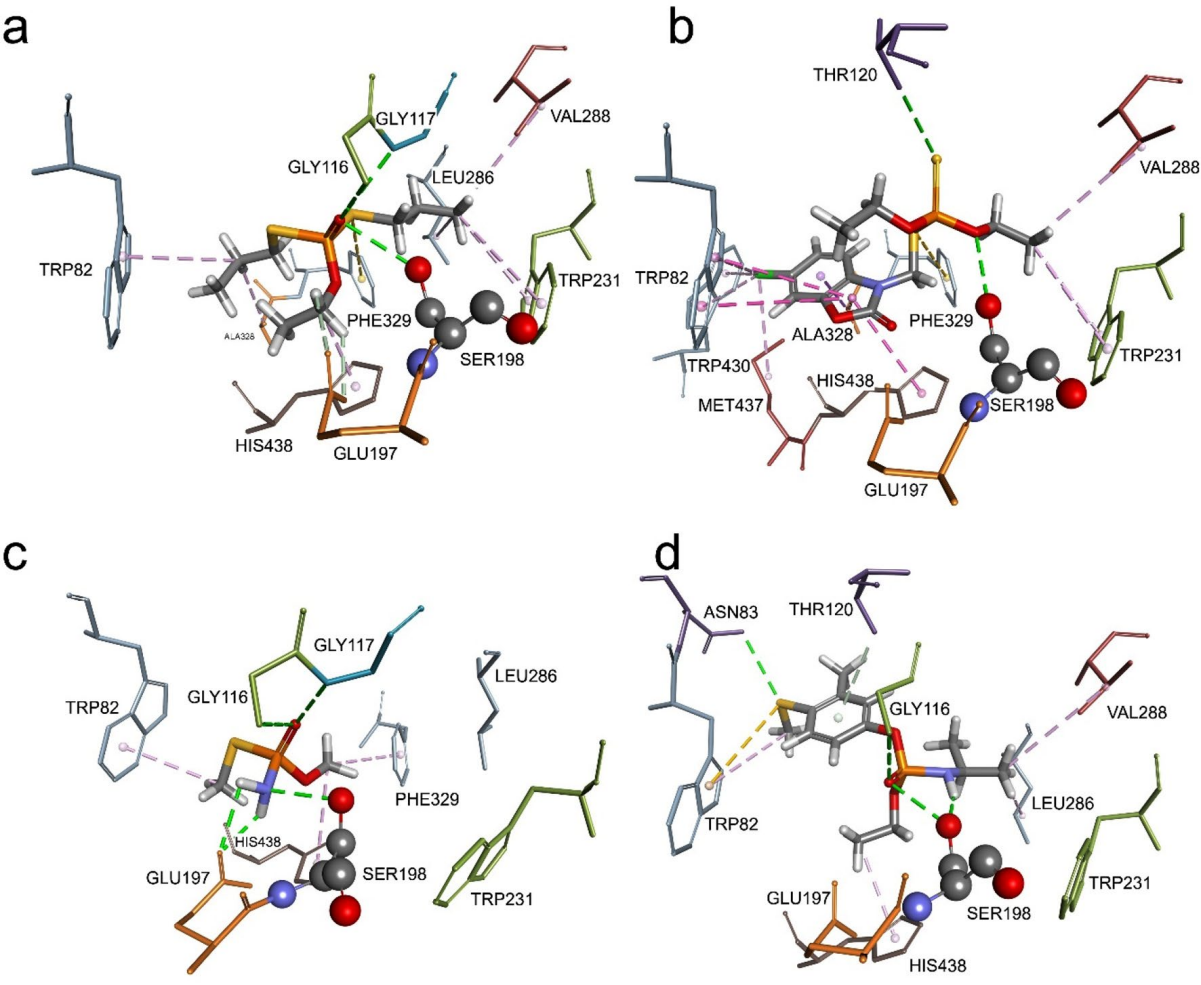
Model of Michaelis–Menten type complex of organophosphorus pesticides: ethoprophos (**a**), phosalone (**b**), methamidophos (**c**), and fenamiphos (**d**) within the active site of hBChE (PDB code: 2PM8^29^). Poses were selected based on criteria that leaving group of pesticide is oriented opposite from oxygen (Oγ) of catalytic serine (Ser198). Pesticide interactions are represented as dashed lines: hydrophobic (purple), hydrogen bonds (green) and electrostatic (orange).

Model of pesticide-BChE complex revealed formation of hydrophobic interactions and hydrogen bonds similar to pesticide-AChE complex (Fig. 4). Due to difference in amino acids residues between BChE and AChE active sites, BChE has about 300 Å larger active site gorge and therefore all pesticides are positioned close to the catalytic Ser198 forming the hydrogen bond with it. Additionally, ethoprophos, methamidophos, and fenamiphos are stabilised in oxyanion hole, forming interaction with Gly116 and/or Gly117. In the case of ethoprophos and phosalone, we noticed simultaneous hydrophobic interactions with Trp82 and Trp231 in the choline binding site and acyl pocket, respectively. This type of interaction is not feasible in the AChE active site, since Phe295 and Phe297 in the acyl pocket block approach of ligands to Trp236.

### Reactivation of inhibited acetylcholinesterase

**Figure 5:**
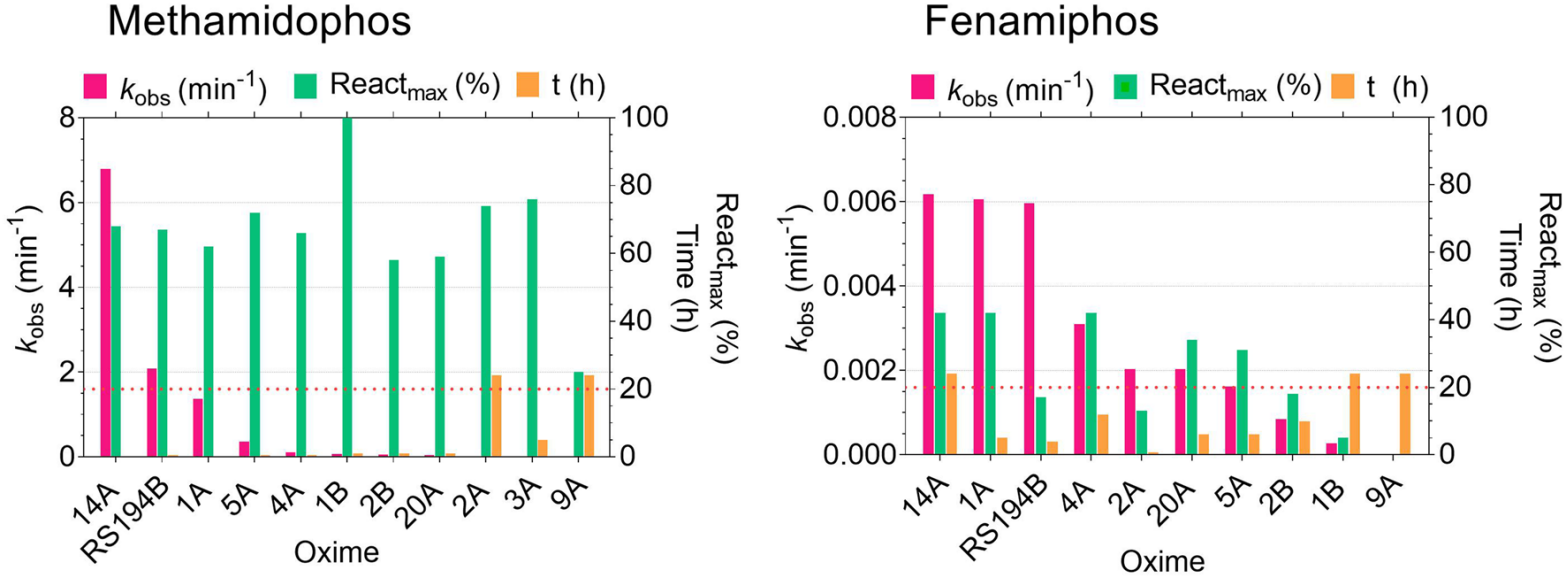
Reactivation screening of pesticide-inhibited hAChE by selected oximes (0.1 mM) measured at 25 °C. Results are expressed as means of the observed reactivation rates (*k*_obs_), maximal reactivation percentages and time to achieve maximum of reactivation determined from at least three experiments.

For evaluation of reactivation kinetics of hAChE we used phosphoroamidates, methamidophos and fenamiphos, as inhibitors, while for reactivators we selected oximes that were ranked as potent antidotes in nerve agent tabun poisoning, which is also phosphoroamidate^21^, and a centrally active oxime RS194B^24^. Oximes were initially monitored for reactivation up to 24 h at 0.1 mM, and potency was rank-ordered in terms of the observed reactivation rate constant (*k*_obs_) as shown in Fig. 5 and Table S1. In general, methamidophos-inhibited hAChE was more susceptible to reactivation than hAChE inhibited by fenamiphos, comparing both *k*_obs_ and percentage of the reactivation maximum. For both pesticides, oximes 14A, 1A and RS194B were most effective reactivators of AChE.

**Figure 6:**
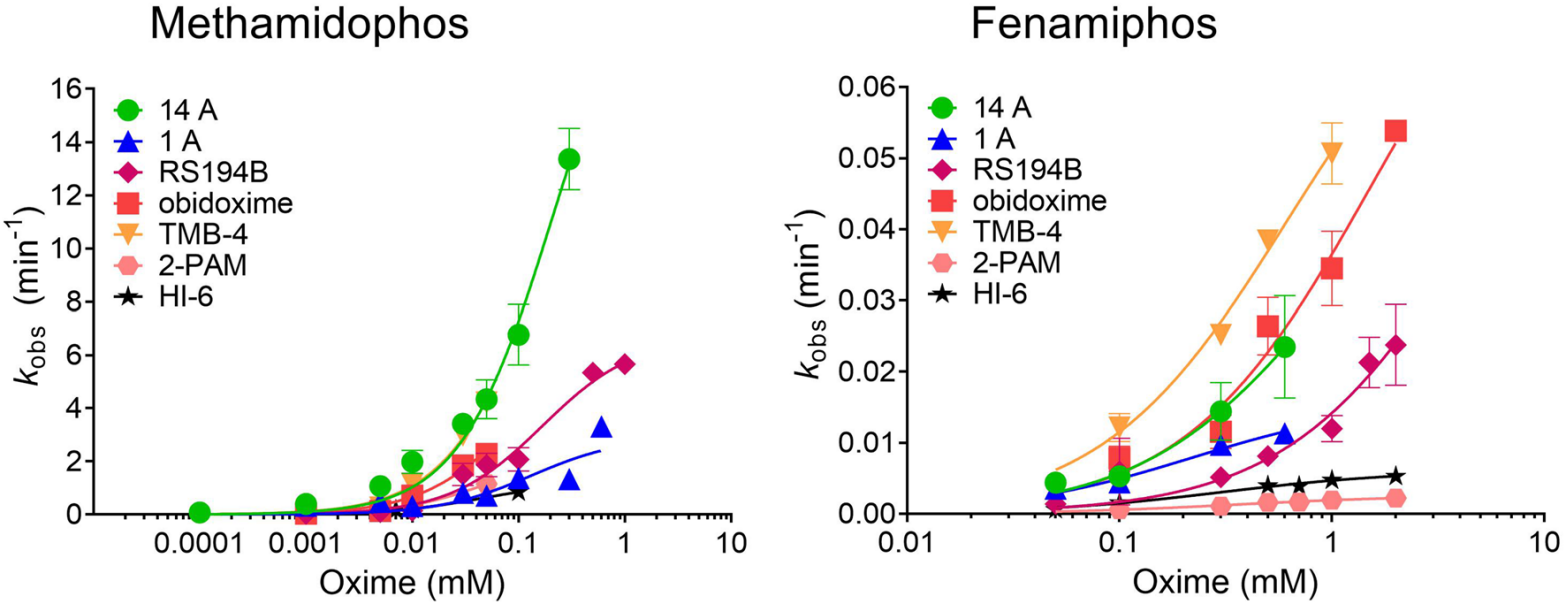
Reactivation kinetics of pesticide-inhibited hAChE by selected oximes. Reactivation constants were determined by nonlinear regression of experimental data and are presented in Table 1. Values are expressed as mean ± SEM from at least three experiments.

Based on the screening results, oximes 14A, 1A and RS194B were chosen for studying reactivation kinetics in detail. The reactivation of hAChE phosphorylated with methamidophos and fenamiphos was evaluated over a wide oxime concentration range (Fig. 6). Four standard oximes: 2-PAM, HI-6, obidoxime and TMB-4, were used for comparison. Reactivation constants were determined, including the maximum reactivation rate constant (*k*_2_) and oxime-enzyme dissociation constant (*K*_OX_) from which the second-order rate constant of reactivation (*k*_r_) was evaluated. Maximal reactivation percentage (React_max_) is also given in Table 2.

**Table 2:** Reactivation of methamidophos- and fenamiphos-inhibited hAChE by selected oximes.

Oxime	*k*_2_ (min^−1^)	*K*_OX_ (mM)	*k*_r_ (min^−1^ M^−1^)	React_max_ (%)
	**Methamidophos**			
1A	2.9 ± 0.6	0.13 ± 0.06	22,400 ± 6,388	40
14A	22.2 ± 3.8	0.20 ± 0.06	108,300 ± 17,090	85
RS194B	6.7 ± 0.9	0.17 ± 0.05	39,810 ± 7,479	100
Obidoxime	6.8 ± 2.3	0.09 ± 0.04	75,320 ± 10,210	70
TMB-4	16.5 ± 6.6	0.14 ± 0.07	116,600 ± 12,380	80
HI-6	1.2 ± 0.1	0.05 ± 0.009	27,140 ± 2,382	80
2-PAM	2.7 ± 0.2	0.06 ± 0.006	42,190 ± 2,547	80
	**Fenamiphos**			
1A	0.016 ± 0.002	0.22 ± 0.06	72 ± 12	40
14A	0.069 ± 0.034	0.81 ± 0.61	85.8 ± 24.8	45
RS194B	0.053 ± 0.019	2.45 ± 1.42	21.5 ± 4.6	45
Obidoxime	0.091 ± 0.026	1.51 ± 0.71	60.7 ± 12.1	65
TMB-4	0.082 ± 0.010	0.61 ± 0.15	134.2 ± 17.6	65
HI-6	0.006 ± 0.001	0.30 ± 0.09	20.3 ± 4.9	65
2-PAM	0.003 ± 0.0002	0.35 ± 0.09	7.5 ± 1.5	70

Kinetic parameters (± SEM): the maximal first-order reactivation rate constant (*k*_2_), the dissociation constant of the phosphylated enzyme-oxime reversible complex (*K*_OX_), overall second-order reactivation rate constant (*k*_r_) and maximal percentage of reactivation (React_max_) were determined from at least three experiments at 25 °C.

In the case of oximes’ aptitude for dephosphylation of the active serine 203, considerable differences between methamidophos and fenamiphos conjugate of hAChE were displayed as could be expected based on different chemical structures (Fig. 1). Methamidophos-inhibited hAChE was reactivated to higher extent than the fenamiphos conjugate despite its reactivation having been monitored for at least 6 h. Some possible explanations could be the formation of stable and reactive phosphorylated oximes, which may re-inhibit reactivated hAChE. Alternatively, the conjugates could undergo dealkylation known as aging that prevents oxime-assisted reactivation^26,27^. Interestingly, all three selected oximes reactivated both conjugates with the same trend—oxime 14A was the most efficient, while RS194B and 1A followed, and this trend is directly in connection to their maximum reactivation rates. In other words, the affinity of hAChE inhibited by methamidophos and fenamiphos towards oximes, embodied by *K*_OX_, did not differ much. Notwithstanding, higher affinity was shown for methamidophos-hAChE conjugate with similar mM range for all tested oximes. Out of standard oximes, TMB-4 and obidoxime were better reactivators of pesticide-inhibited hAChE than HI-6 and 2-PAM. Due to fast reactivation rate, oximes 14A and TMB-4 exhibited the most efficient hAChE reactivation in case of both OP pesticides, while RS194B was effective in reactivation of methamidophos-inhibited AChE.

### Molecular modelling of reactivation complex for methamidophos-hAChE conjugate with selected oximes

**Figure 7:**
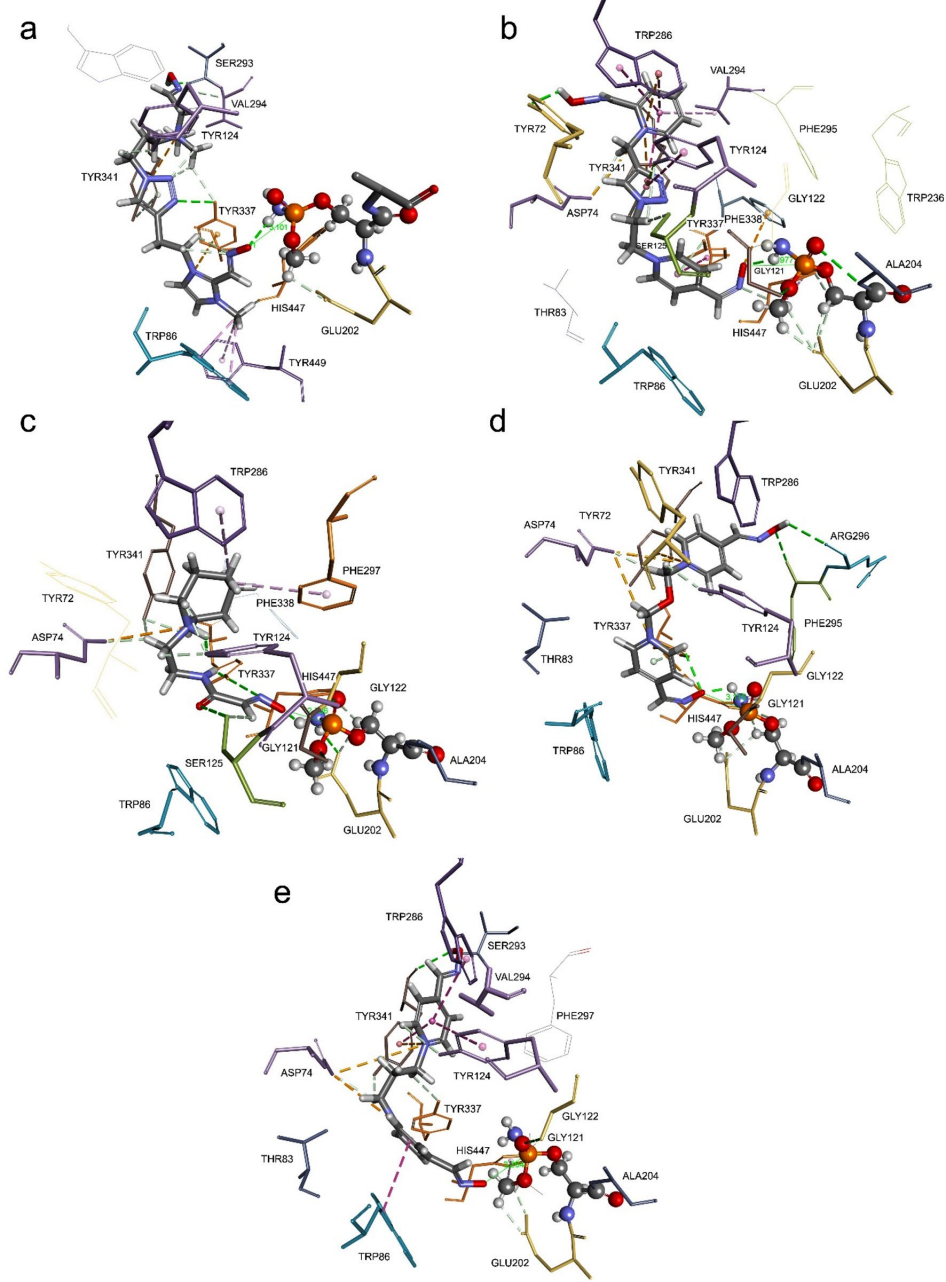
Model of reversible complex between oxime reactivator and methamidophos hAChE conjugate. Docked compounds are: 1A (**a**), 14A (**b**), RS194B (**c**), obidoxime (**d**), and TMB-4 (**e**). The catalytic Ser203-methamidophos conjugate is presented as a stick and ball. Interactions with amino acid residues are represented as dashed lines: hydrophobic (purple), hydrogen bonds (green) and electrostatic (orange).

We simulated in silico the Michaelis-type complex between oxime reactivator and conjugated human AChE with methamidophos (Fig. 7). Molecular docking generated positions of oxime reactivator within 5 Å from methamidophos phosphorus atom. Deprotonated O atom of the oxime group was in line with P and Oγ atom from catalytic Ser203, since according to SN_2_ mechanism of reactivation, this orientation of atoms will generate a transition state^28^. Distance between deprotonated O atom of oxime group and phosphorus atom of methamidophos were 3.10 Å, 3.97 Å, 2.71 Å, 3.11 Å and 3.26 Å, for 1A, 14A, RS194B, obidoxime and TMB-4, respectively. Selected oximes are stabilised mainly via hydrophobic interactions at the peripheral anionic site (Tyr72, Tyr124, Trp286) and at the choline binding site (Trp86 and Tyr337) (Table S2). Interestingly, oximes 14A, RS194B and obidoxime do not create hydrophobic interaction with Trp86. Interactions of 14A at the peripheral anionic site are somewhat different from obidoxime and TMB-4 probably due to larger linker containing triazole ring (Fig. 7). One pyridinium ring of 14A is stabilized at the peripheral anionic site via hydrophobic interactions with Tyr124, Trp286 including Tyr341 and Val294, triazole ring is stabilised with Tyr124, Tyr341 and Ser125, while the second pyridinium ring of 14A is stabilised at the catalytic site with Tyr337 and Phe338. Interestingly, N atom from tertiary amine of RS194B overlaps with N atom from pyridinium rings of obidoxime and TMB-4, while the 2-hydroxyiminoacetamide group is stabilized via multiple H-bonds from Ser125 and Tyr337.

Since these complexes between oxime reactivator and methamidophos hAChE conjugate were generated by molecular replacement, we performed molecular dynamics simulation (20 ns) to monitor changes in distance between deprotonated O atom of an oxime group from P atom of methamidophos-hAChE conjugate (Figure S1). Molecular dynamics showed that distances were enlarged due to conformational changes of active site residues over time (Figure S2). Nevertheless, changes in those distances were stabile during 20 ns simulation indicating stabile binding of oximes within AChE active site. However, more favourable interactions with residues resulted with non-favourable position of deprotonated O atom of the oxime group for reactivation, as we expected. Generally, binding of oxime reactivator is more favourable within the apo-AChE than in AChE inhibited by OP.

## Discussion

Our kinetic study characterised inhibition of human AChE and BChE with four OP pesticides. Inhibitory potency was superior for hAChE in comparison to hBChE, with exception of fenamiphos. The observed selectivity of ChEs inhibition is a direct consequence of divergence in active site gorge between these two enzymes, as the human BChE active site differs in six aromatic residues found in AChE, leading to larger active site^28–31^. Previous studies showed that bulkier molecules have higher inhibition rate for BChE than AChE because of easier access to the catalytic gorge^32–35^. Similar trend in this case is observed for phosphoroamidates, where hBChE has almost 15-fold higher affinity for fenamiphos than hAChE. On the other hand, methamidophos was less prominent inhibitor for both enzymes, especially hBChE, probably due to difficulty of smaller molecule to achieve optimal positioning in its spacious active gorge. In addition, phosphoramidates with O-methyl group were generally less potent inhibitors of hAChE compared to O-ethyl analogues^14^.

Out of four tested pesticides ethoprophos was the most potent inhibitor of hAChE and it was 4 times more effective than in BChE inhibition. The high inhibition potency of hAChE could be a consequence of its symmetry and two thiopropyl substituents that may each act as the leaving group. On the other hand, low potential of phosalone to inhibit hAChE could be also due to its leaving group—condensed bicyclic heteroatomic ring, which can create multiple hydrophobic interactions with active site aromatic residues that can limit its orientation in vicinity of the catalytic serine. It is worth mentioning that phosalone as thio-phosphate is less toxic derivative of pesticides than its oxo-phosphate analogue formed *in vivo*^12,17^.

Previous studies have shown that phosphoroamidate tabun is notoriously resistant toward reactivation by certain oximes, probably as a result of electron pair located on tabun’s amidic group making the nucleophilic attack by oxime very difficult^36,37^. To test whether this premise includes other phosphoramidates, we conducted experiments on reactivation of hAChE inhibited by methamidophos and fenamiphos. Most efficient reactivators of tabun were shown to be quaternary oximes^38–42^ and related tendency was observed in screening reactivation of fenamiphos and methamidophos inhibited-hAChE. Oximes 14A, obidoxime and TMB-4 displayed the highest potency in removal of conjugated phosphoryl moiety from the active serine, confirming previous reports that the *para*- positioned oxime group provides more flexible approach and achieves stabilization in vicinity of tabun-phosphorylated serine^26,39,43^. Oxime 14A with *para*- and *ortho*-positioned oxime groups showed the highest efficacy in reactivation of unbranched methamidophos, reactivating hAChE to 80% in 1 min. Fenamiphos, on the other hand, as a phosphoroamidate with substituted amino group, same as tabun, was similarly resistant to reactivation^21^. This confirms again that reactivation efficacy depends on structure of inhibitor, as does the structure of reactivator.

In addition, hydroxyiminoacetamido amine oxime RS194B, previously identified as simultaneous CNS and peripheral nervous system reactivator^22–25,44,45^, showed a high capability for the nucleophilic displacement to reactivate both methamidophos and fenamiphos. In comparison to the standard bispyridinium oximes, it seems that overall reactivation capability of RS194B is limited by lower binding affinity as one could expect for non-permanently charged ligand. Previous study on tabun reactivation with RS194B analogues has highlighted imidazole oximes^46^, although they still fall short of bispyridinium oximes^38,40,42^. This implies that structure of oxime, as well as geometry of oxime approach to the phosphorus atom conjugated to the active serine, are important criteria for efficient reactivation along with the chemical nature of the phosphorus moiety^47^. For efficient reactivation of AChE inhibited with phosphoroamidate tabun, oximes should have a five- or six-atom linker between oxime group and the aromatic part of the molecule, which is consistent with the analysed zwitterionic reactivators^46^. This study shows that RS194B is a much better reactivator of methamidophos- or fenamiphos-inhibited hAChE than 2-PAM and HI-6 and comparable to obidoxime. While previous studies with OP nerve agents-inhibited AChE sorted RS194B as a lead in a family of ionizable, zwitterionic oximes, our research provides evidence that RS194B also exhibits substantial universality to be an effective reactivator of pesticide-inhibited AChE.

## Conclusions

Our findings offer a valuable kinetic data on inhibition of both cholinesterases with four OP pesticides and reactivation of inhibited hAChE. Structure of pesticides governs inhibition specificity and selectivity, similarly as for the reactivation process. It is clearly showed that two newly developed reactivators—bispyridinium triazole oxime 14A and zwitterionic oxime RS194B – possess remarkable potential for further development of antidotes against OP pesticide poisonings.

## Materials and Methods

### Chemicals and enzymes

Organophosphorus compounds (OPs) methamidophos, fenamiphos, phosalone and ethoprophos were purchased from Sigma-Aldrich (St. Louis, MO, USA). Stock solutions of pesticides (5000 μg/mL) were made in isopropyl alcohol and were stored at 4 °C. Further dilutions were made in water before the measurement.

Majority of used oximes, as well as RS194B were generous gifts from Prof Palmer Taylor (Skaggs School of Pharmacy and Pharmaceutical Sciences, University of California at San Diego, La Jolla, United States). Oximes HI-6 and 2-PAM were purchased from Sigma-Aldrich (St. Louis, MO, USA). Obidoxime and TMB-4 were a generous gift from Dr Kamil Kuča (University of Hradec Kralove, Hradec Králové, Czech Republic). Oximes were dissolved in water at starting 100 mM or 10 mM concentration and stored at 4 °C. Reagents 5,5-dithiobis-(2-nitrobenzoic acid), acetylthiocholine (ATCh), and bovine serum albumin (BSA) were purchased from Sigma-Aldrich (St. Louis, MO, USA) and diluted in sodium phosphate buffer (0.1 mM, pH 7.4), while ATCh was diluted in water just before use.

Recombinant human acetylcholinesterase (hAChE) was prepared as described previously^48^ and was a gift from Prof Palmer Taylor and Dr Zoran Radić (Skaggs School of Pharmacy and Pharmaceutical Sciences, University of California at San Diego, La Jolla, United States). hAChE was diluted in 1% of BSA buffer as work solution and stored at 4 °C. hBChE derived from purified human plasma was a generous gift from Dr Florian Nachon (*Département de Toxicologie et Risques Chimiques, Institut de Recherche Biomédicale des Armées*, Bretigny-sur-Orge, France).

### Cholinesterase kinetic activity measurements

Inhibition of hAChE and hBChE was measured at designated times (up to 60 min) with organophosphorus pesticides (0.1–100 µM). Enzyme activity was measured upon addition of ATCh (1 mM) using Ellman method^49^ at 25 °C and 412 nm, on a CARY 300 spectrophotometer (Varian Inc., Australia) with temperature controller as described previously^34^. The inhibition rate constants at a given pesticide concentration (*k*_obs_) were calculated by linear regression analysis, while a wider OP concentration range enabled the determination of inhibition constants as described previously^32^. Data was expressed as mean ± SEM with each experiment preformed at least as a triplicate and calculated using Prism7 software (GraphPad, San Diego, USA).

For reactivation experiments, hAChE was incubated with OPs for about 60 min until up to 95–100% inhibition was achieved. The incubation mixture was then filtrated through a Sephadex G-50 spin column (Roche Diagnostic GmbH, Mannheim, Germany) to remove the excess of unconjugated OP. After gel filtration by centrifugation, enzyme was diluted tenfold in 0.1 M sodium phosphate buffer, pH 7.4, containing 0.01% BSA and incubated with oxime at 25 °C. After a defined reactivation time, aliquots were taken and diluted 100-fold for measurement of the enzyme activity by the Ellman method on a CARY 300 spectrophotometer (Varian Inc., Australia) with temperature controller^49^. Detailed reactivation kinetics using a wider oxime concentration range enabled the determination of reactivation constants: *k*_2_ (maximal first-order reactivation rate constant), *K*_OX_ (apparent phosphylated enzyme-oxime dissociation constant) and *k*_r_ (overall second-order reactivation rate constant). *k*_2_ and *K*_OX_ were evaluated from the plot *k*_obs_ vs oxime concentration as previously described with slight modifications^47^. At each oxime concentration, *k*_obs_ was calculated from linear regression analysis:

1 $${\text{ln }}\left( {v_{0} - v_{{\text{t}}} /v_{0} } \right)\, = \,{\text{t }} \cdot k_{{{\text{obs}}}}$$

and multiplied with 0.01x maximal reactivation percentage of given concentration to equalize it for each concentration; maximum reactivation rate constant (*k*_2_) and oxime-enzyme dissociation constant (*K*_OX_) were obtained by the non-linear fit:

2 $$k_{{{\text{obs}}}} \, = \,k_{{2}} \left[ {{\text{OX}}} \right] / \left( {K_{{{\text{OX}}}} \, + \,\left[ {{\text{OX}}} \right]} \right)$$

while the second-order rate constant of reactivation (*k*_r_) was their ratio:

3 $$k_{{\text{r}}} \, = \,k_{2} /K_{{{\text{OX}}}}$$

### Molecular modelling

The compounds to be docked in the active site of hAChE and hBChE were modelled and later minimized with the MMFF94 force field using ChemBio3D Ultra 12.0 (PerkinElmer, Inc., Waltham, MA, USA). The Discovery Studio 20.1 (BioVia, San Diego, CA, USA) Dock Ligands protocol (CDOCKER) with a CHARMm force field was used for the docking study^50,51^. As a model of hAChE we used crystal structure of human AChE (PDB code 4PQE^52^). The model of hBChE was the crystal structure of BChE (PDB code 2PM8)^30^. The binding site within the hAChE and hBChE was defined by a sphere (r = 13 Å) and it was used as the rigid receptor^53^. Details about docking procedure using the CDOCKER protocol and scoring of generated ligand poses by a CHARMm energy were described previously^51^.

Molecular modelling was performed using the BioVia Discovery Studio 20.1 and CHARMm based CDOCKER docking protocol. As a model of methamidophos inhibited human AChE we used crystal structure of human AChE (PDB code 4PQE^52^) and the crystal structure of mouse AChE inhibited with fenamiphos (PDB code 2WU3^54^). Molecular replacement of conjugated Ser203 from 2WU3 into 4PQE was made after superposition of 4PQE and 2WU3 structures. Structure of methamidophos conjugate was created by deleting methyl group and isopropyl group from fenamiphos conjugate. In the next step the reactivation product, a phosphylated oxime reactivator, was docked into a Ser203Gly mutant of 4PQE structure. Ser203Gly mutant was created to enlarge space of AChE catalytic site and enable better positioning of conjugate of methamidophos and oxime reactivator. Obtained poses were analysed and ones with methamidophos moiety positioned in the oxyanion hole were selected and transferred into the structure of methamidophos human AChE conjugate. Methamidophos moiety was deleted from oxime reactivator and oxygen atom from oxime group was deprotonated, which is consistent to SN_2_ mechanism of reactivation. Complex of oxime reactivator and methamidophos human AChE conjugate was fully minimised using CHARMm forcefield and RMS gradient tolerance was set to 0.01 using the BioVia Discovery Studio 20.1.

Molecular dynamics simulation was performed using Standard Dynamics Cascade protocol in Discovery studio 20.1. Protocol includes following steps cascade: full system minimisation using Steepest Descent algorithm with RMS gradient set to 1.0, full system minimisation using Adopted Basis NR algorithm with RMS gradient set to 0.1, heating step for 4 ps T = 310 K, equilibration step for 20 ps T = 310 K and production step for 20 ns and target temperature T = 310 K.

## Supplementary Information

Supplementary Information.
