# Supplementary material for: Assessment of four organophosphorus pesticides as inhibitors of human acetylcholinesterase and butyrylcholinesterase
Source: Sci Rep. 2021 Nov 2;11:21486. doi: 10.1038/s41598-021-00953-9 (PMC8563940; doi:10.1038/s41598-021-00953-9)
Supplement: Supplementary file 1 — Supplementary Information. [file 41598_2021_953_MOESM1_ESM.pdf]

Scientific Reports, 2021, https://doi.org/10.1038/s41598-021-00953-9

Supplementary material

Assessment of four organophosphorus pesticides as inhibitors of human acetylcholinesterase and butyrylcholinesterase

Tena Čadež, Dora Kolić, Goran Šinko and Zrinka Kovarik*

Institute for Medical Research and Occupational Health, Ksaverska cesta 2, HR-10 000 Zagreb, Croatia
*Correspondence: Z Kovarik (zkovarik@imi.hr; + 3851 4682 555)

Table S1. Reactivation of pesticide-inhibited human AChE with selected oximes (0.1 mM). Obidoxime, TMB-4 and 2-PAM were 0.05 mM in reactvation of methamidophos-hAChE conjugates. Inhibition (%) of AChE with 1 µM oxime is also given.

Oxime	Oxime structure	Inhibition %	Methamidophos			Fenamiphos		
			k_{obs} / min $^{-1}$	*React*$_{max}$/ %	t / h	k_{obs} / min $^{-1}$	React$_{max}$/ %	t / h
1A	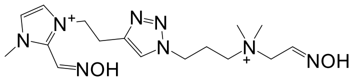	13	1.3721	62	0.16	0.0061	42	5
2A	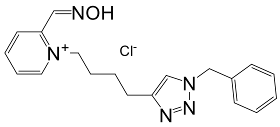	10	0.0159	74	24	0.0020	13	24
3A	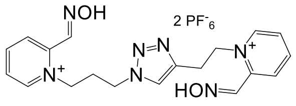	30	0.0094	76	5	0.0023	9	10
4A	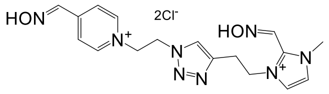	24	0.1021	66	0.5	0.0031	42	12
5A	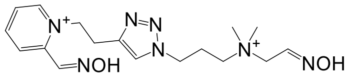	9	0.3533	72	0.5	0.0016	31	6
9A	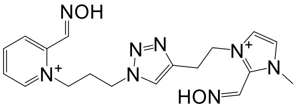	63	0.0013	25	24	0.0000	0	24
14A	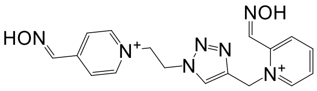	10	6.7944	68	0.08	0.0062	42	3
20A	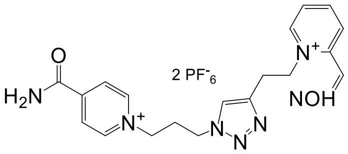	23	0.0352	59	1	0.0020	34	6
1B	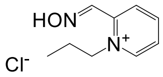	6	0.0623	100	1	0.0003	5	24
2B	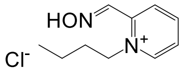	3	0.0580	58	1	0.0008	18	10
RS194B	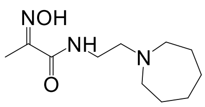	9	2.0772	67	0.5	0.0059	17	4
Obidoxime	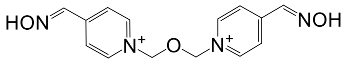	10	2.2807	86	0.16	0.0081	48	3.5
TMB-4	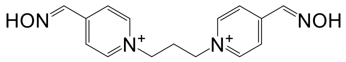	8	4.2338	83	0.16	0.0177	49	1
HI-6	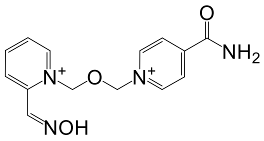	13	0.8518	63	0.19	0.0020	58	20
2-PAM	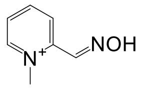	11	1.1558	86	0.36	0.0011	46	20

Table S2. Oxime reactivator interactions with AChE active site residues (Figure 7).

Oxime	Hydrogen bond	Electrostatic	Hydrophobic
1A	Ser293	Tyr337	Trp86
	Val294	Tyr341	Tyr449
	Tyr337		
	Tyr341		
	His447		
14A	Asp74	Asp74	Tyr124
	Tyr124	Tyr286	Trp286
	Ser125	Phe338	Val294
	Tyr337	Tyr341	Tyr337
	Tyr341		Tyr341
RS194B	Asp74	Asp74	Trp286
	Tyr124		Phe297
	Ser125		
	Tyr337		
	Tyr341		
Obidoxime	Asp74	Asp74	–
	Tyr124	Tyr341	
	Phe295		
	Arg296		
	Tyr337		
TMB-4	Asp74	Asp74	Trp86
	Tyr124		Tyr124
	Ser293		Trp286
	Tyr337		Tyr341
	Tyr341		

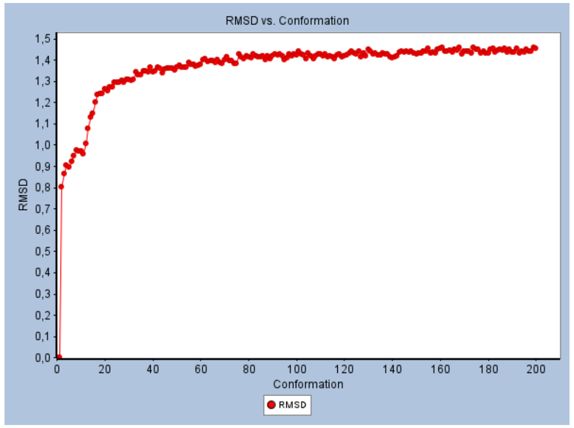

1A

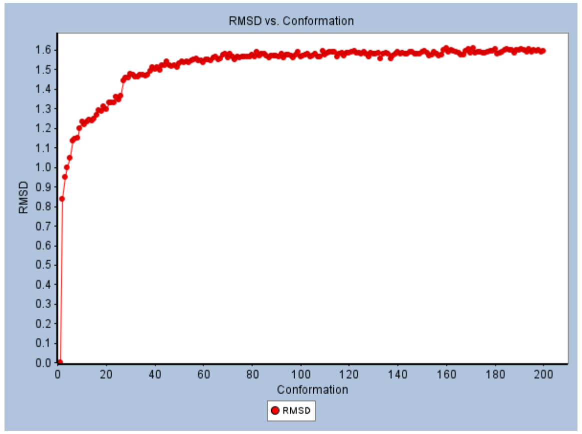

14A

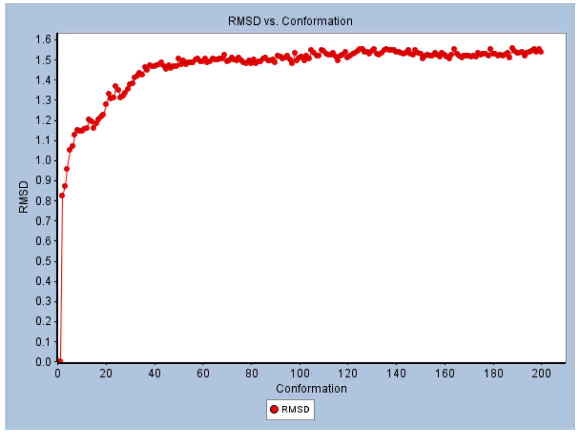

RS194B

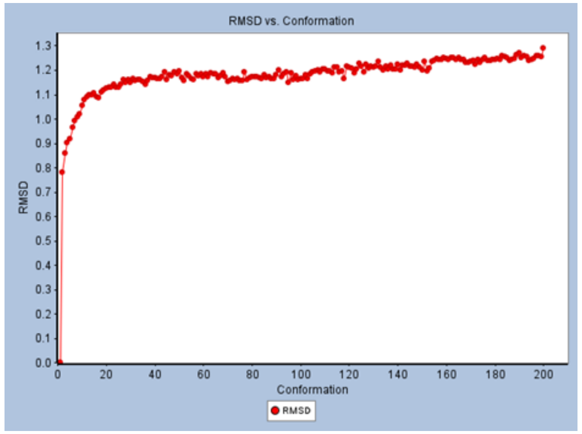

TMB-4

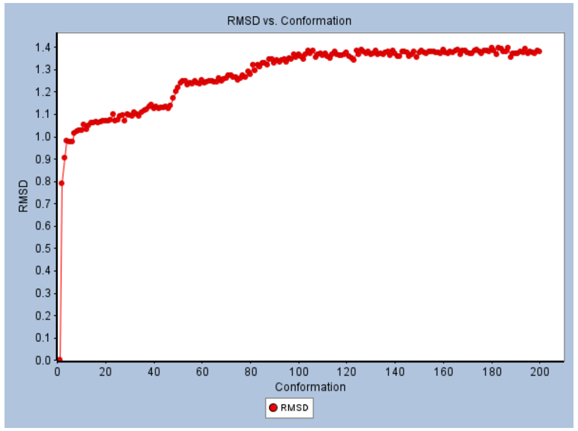

Obidoxime

Figure S1. Change in RMSD value of modelled system during molecular dynamics of oxime in the AChE − methamidophos conjugate, t = 20 ns.

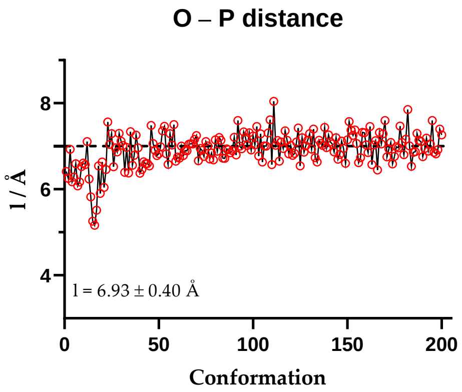

1A

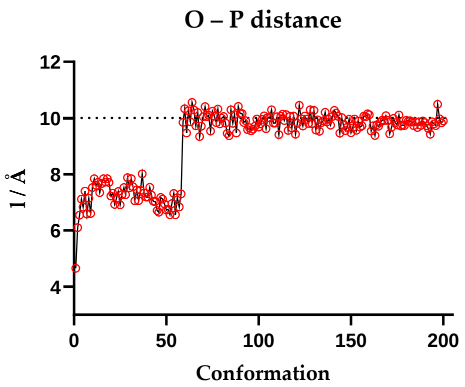

14A

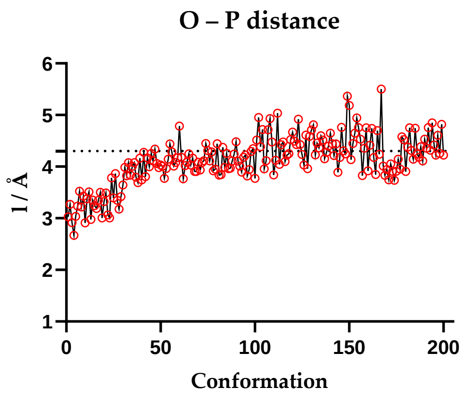

RS194B

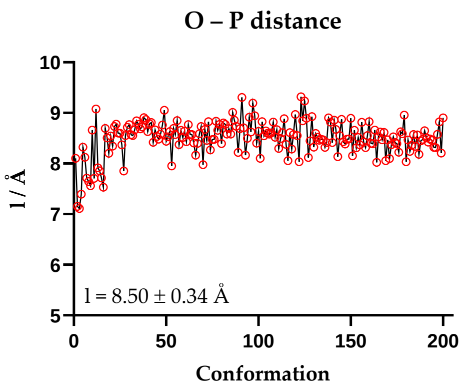

TMB-4

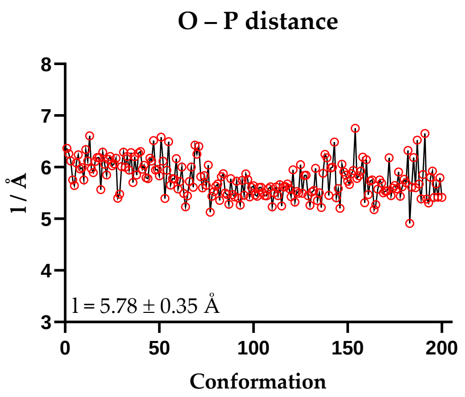

Obidoxime

Figure S2. Change in distance (l) between deprotonated O atom of the oxime group and P atom in the AChE − Methamidophos conjugate. When standard deviation of *l* was less than 10% of mean, average value is reported. Duration of molecular dynamics of system was t = 20 ns.
